# IS THERE AN ASSOCIATION BETWEEN OBSTRUCTIVE SLEEP APNEA AND FRAILTY IN OLDER VETERANS WITH DIABETES?

**DOI:** 10.1093/geroni/igz038.3447

**Published:** 2019-11-08

**Authors:** Jahan E Mahjabin, Dhanya Baskaran, Christopher Lewis, Valeria C Baldivieso, William Wohlgemuth, Jorge G Ruiz

**Affiliations:** 1 University of Miami Miller School of Medicine, Miami, Florida, United States; 2 Miami VA, Miami, Florida, United States; 3 Jackson Memorial Hospital, Miami, Florida, United States; 4 Miami VAHS Geriatric Research, Education and Clinical Center, Miami, Florida, United States

## Abstract

Obstructive Sleep Apnea is a highly prevalent disease, where incidence increases with age. Individuals with chronic diseases such as diabetes and obesity are at risk of OSA increasing the risk of frailty. A retrospective chart review was conducted to study the association between OSA and frailty in older diabetic Veterans. Baseline polysomnography data for 91 patients ≥ 65 years was obtained from the electronic health records at the Miami VA Medical Center. Patients were screened for frailty from January 2016 to August 2017, and followed until October 2018. Patients were then dichotomized into frail (Frailty Index (FI) ≥.21) and non-frail (robust FI =<.10 and pre-frail FI ≥.10, <.21) groups. The mean participant age is 70.9 years, with (SD) of 4.8. The mean age for the frail group is 71.1 years, with a SD of 5.2. Mean age for the non-frail group is 70.5 years, with a SD of 4.2. Linear regression demonstrated a significant positive linear relationship between BMI (t=2.096 p-value= .039) and the frailty index. In binomial logistic regression, adjusting for covariates, BMI was associated with increased apnea severity (OR=1.139, 95% CI= 1.044-1.241), p=.003. However, no significant association was found between FI and apnea severity. The severity of OSA based on the Apnea-Hypopnea Index had no significant association with frailty status. However, the study demonstrated a significant association between obesity and frailty, where higher BMI coincided with higher frailty. Increasing BMI coincided with increased severity of OSA, suggesting that BMI acts as a possible confounder between frailty and OSA.

